# Supplementary figures and images for: Antibiotics modulate attractive interactions in bacterial colonies affecting survivability under combined treatment
Source: PLoS Pathog. 2021 Feb 1;17(2):e1009251. doi: 10.1371/journal.ppat.1009251 (PMC7877761; doi:10.1371/journal.ppat.1009251)

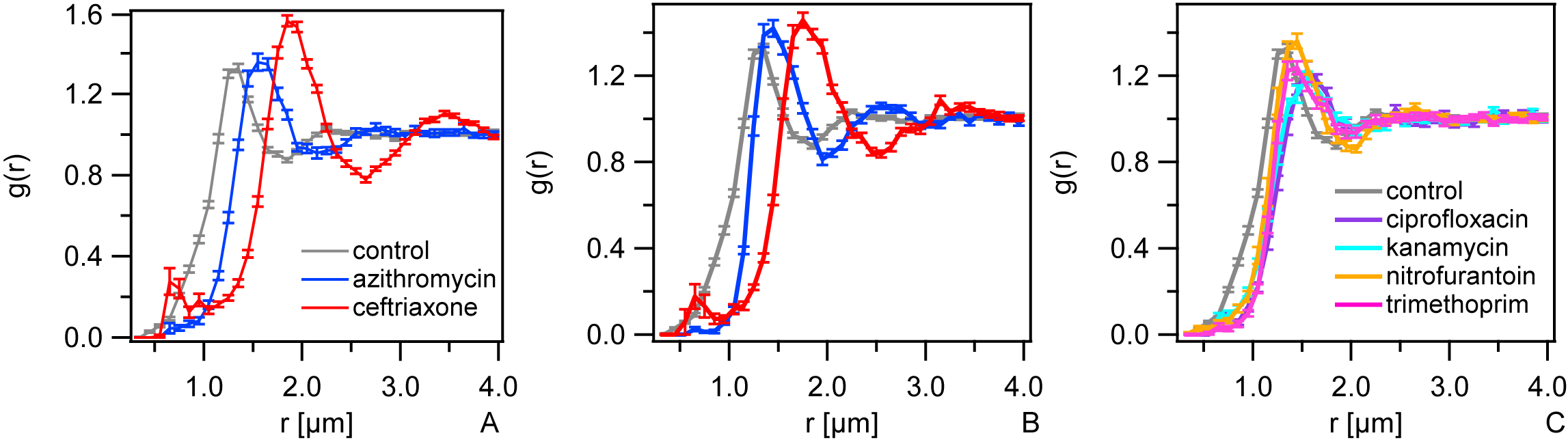

Supplement: S1 Fig — Bacteria (wt*, Ng150) were inoculated into flow chambers and colonies were allowed to assemble for 1 h. Subsequently, they were treated with antibiotics for 3 h at A) 100x MIC, or B, C) 1x MIC. The values for r0 shown in Fig 2D–2F are obtained from fits to Eq 1 to these distributions. (TIF) [file ppat.1009251.s001.tif]

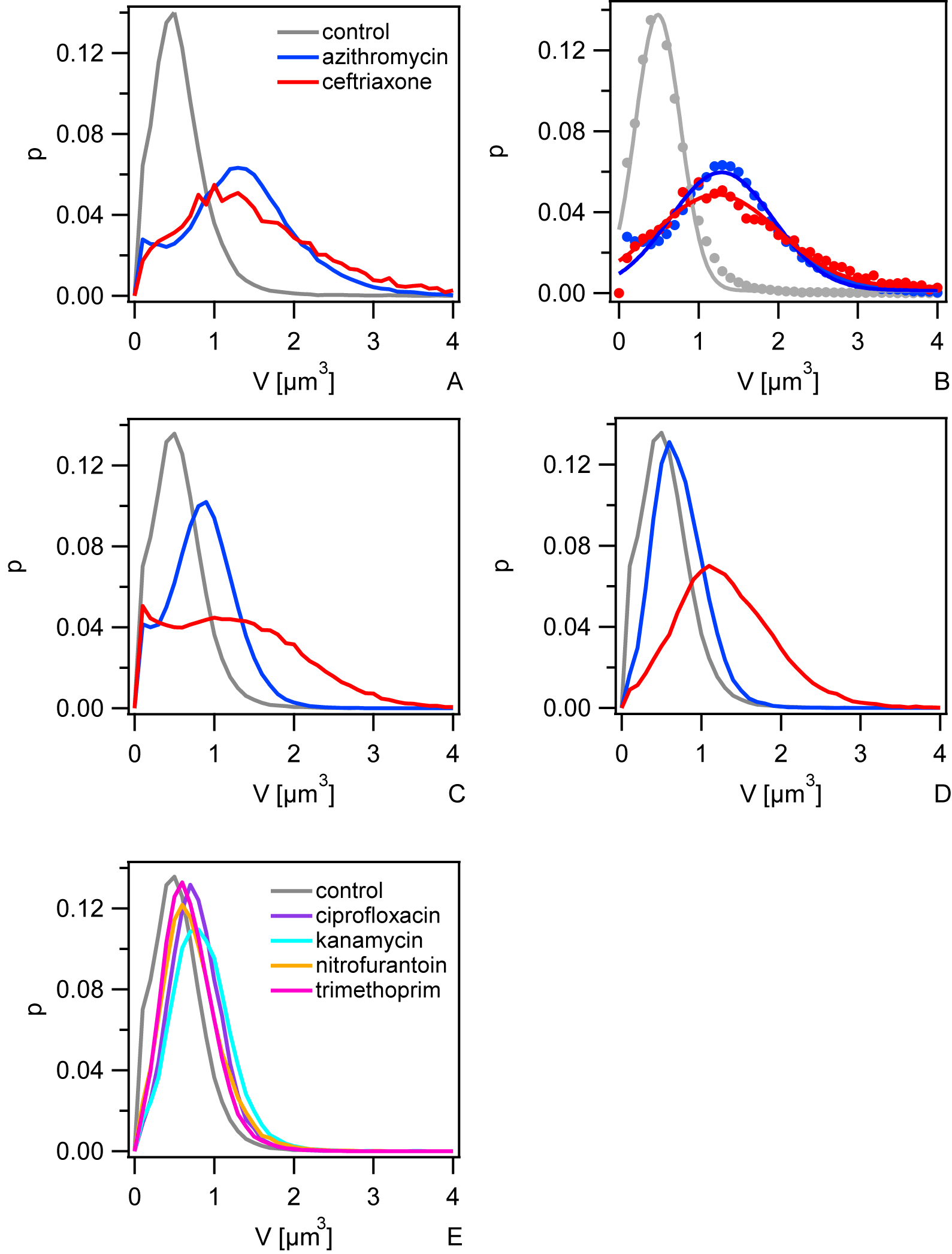

Supplement: S2 Fig — Bacteria (wt*, Ng150) were inoculated into flow chambers and colonies were allowed to assemble for 1 h. Subsequently, they were treated with antibiotics for A, B) 5 h at 100x MIC, C) 3 h at 100x MIC, D—E) 3 h at 1x MIC. The mean values shown in Fig 2E–2G are obtained from Gaussian fits to these distributions. B) Markers: data points, full lines: fits to Gaussian function. (TIF) [file ppat.1009251.s002.tif]

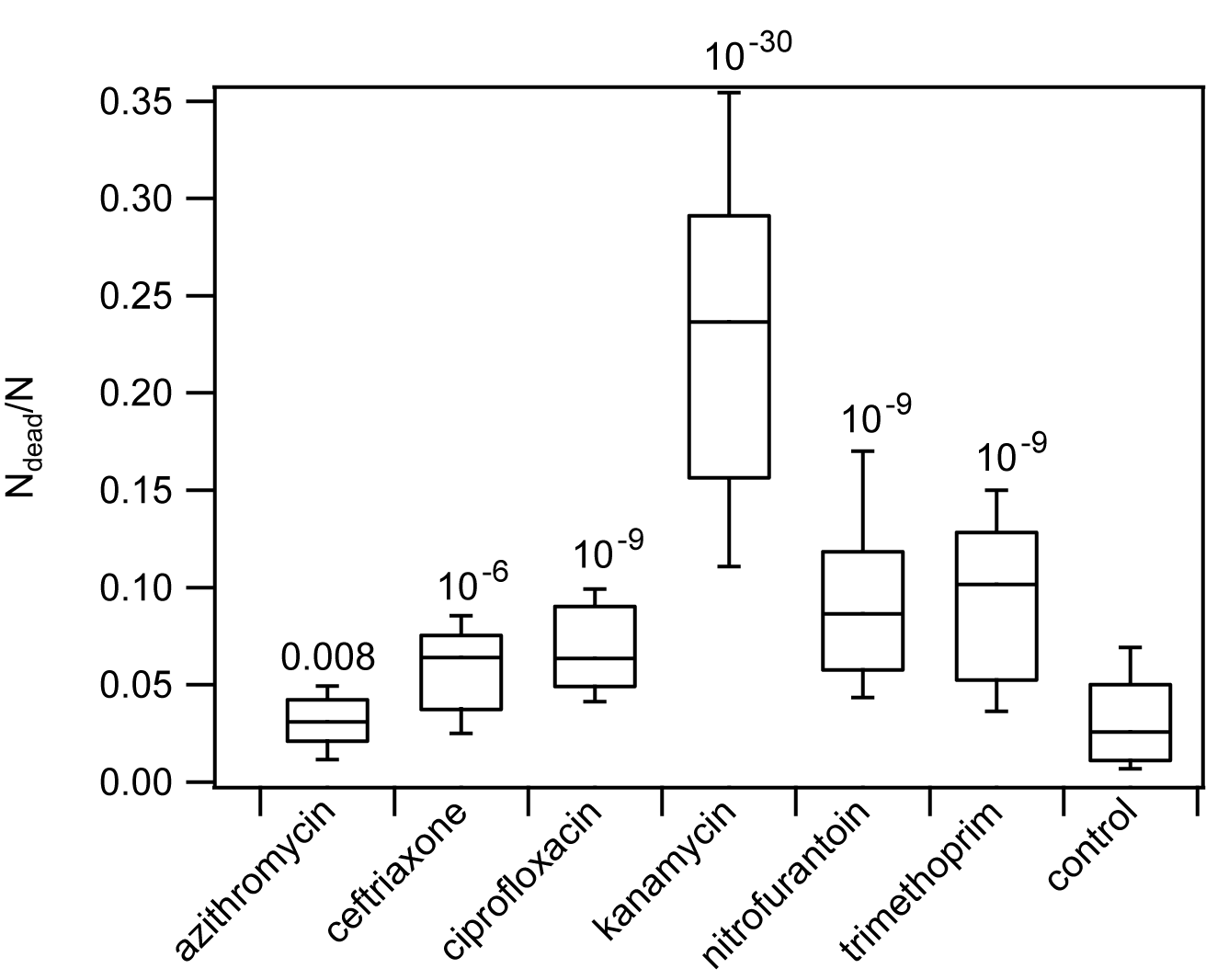

Supplement: S3 Fig — Numbers: p-values from two sample KS-test against the control. (TIF) [file ppat.1009251.s003.tif]

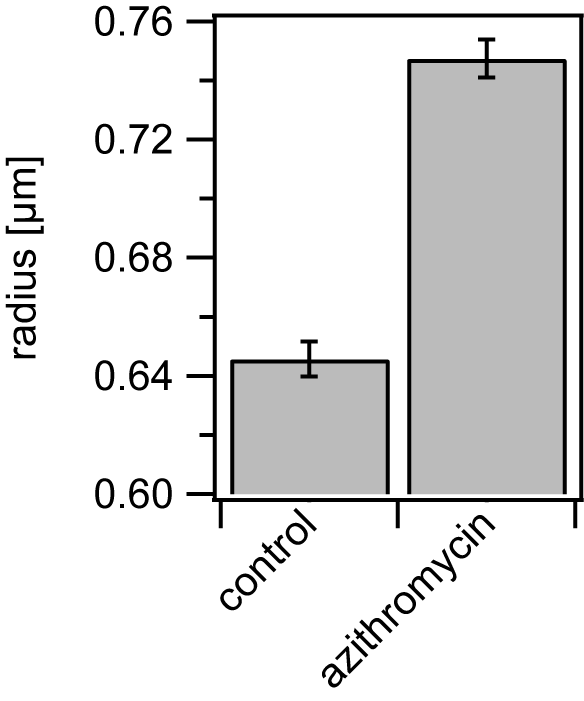

Supplement: S4 Fig — Wt* cells (Ng150) were incubated for 1 h in liquid medium and subsequently treated with azithromycin at 100x MIC for 2 h. Radii of individual cells were determined using phase contrast microscopy. Mean ± standard error of radii of N = 75 cells. p < 10−16 (KS test). (TIF) [file ppat.1009251.s004.tif]

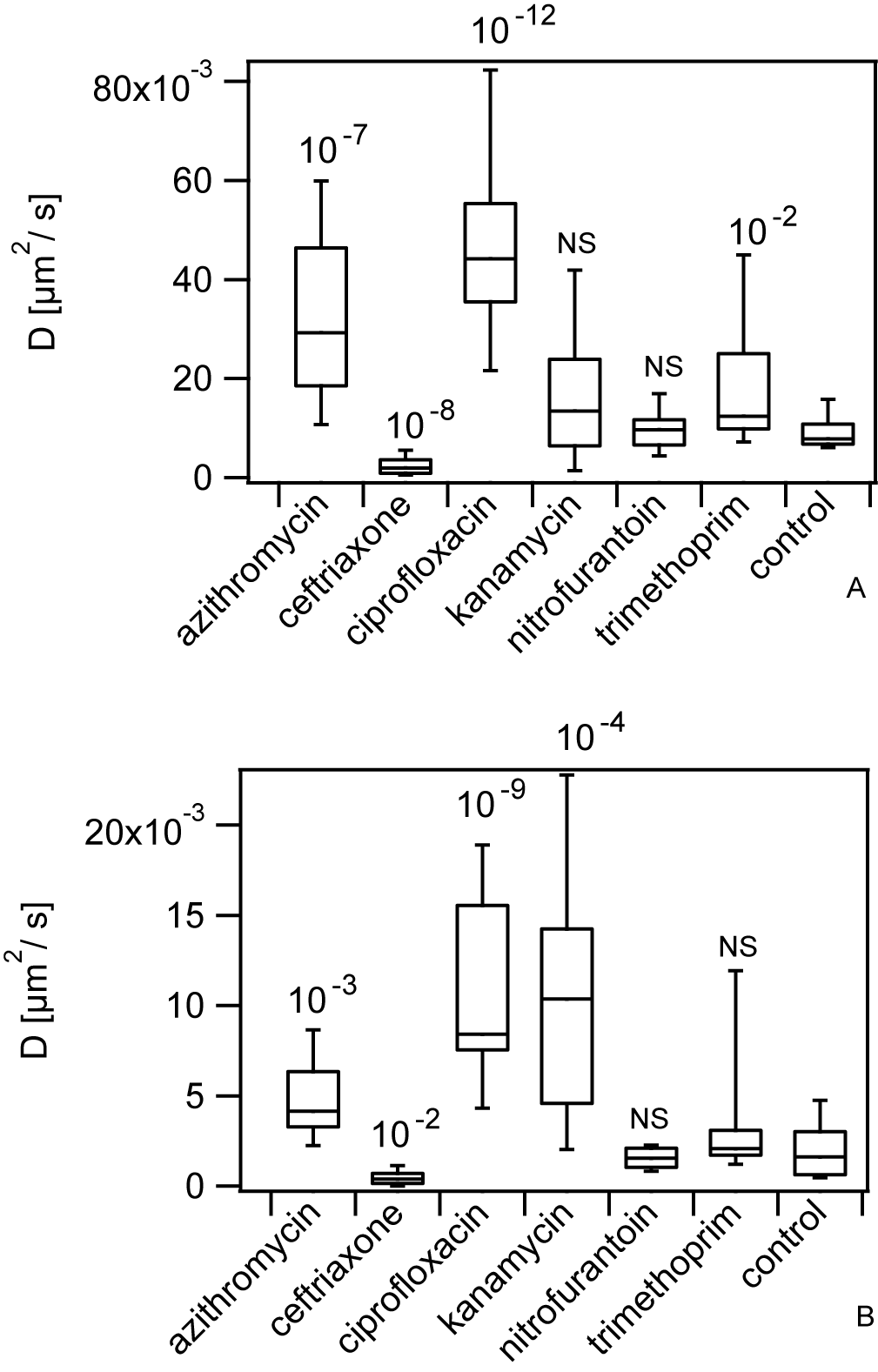

Supplement: S5 Fig — wt* gonococci (Ng150) were inoculated into flow chambers and colonies were allowed to assemble for 1 h. Subsequently, they were treated with different antibiotics at their respective MICs for 3 h (0.064 μg / ml azithromycin, 0.004 μg / ml ceftriaxone, 0.002 μg / ml ciprofloxacin, 20 μg / ml kanamycin, 0.48 μg / ml nitrofurantoin, 32 μg / ml trimethoprim). Effective diffusion constant was measured at A) the edge and B) at R = 5 μm into the colony. N = 12–20 colonies. Numbers are p-values from two sample KS-test against the respective controls. (TIF) [file ppat.1009251.s005.tif]

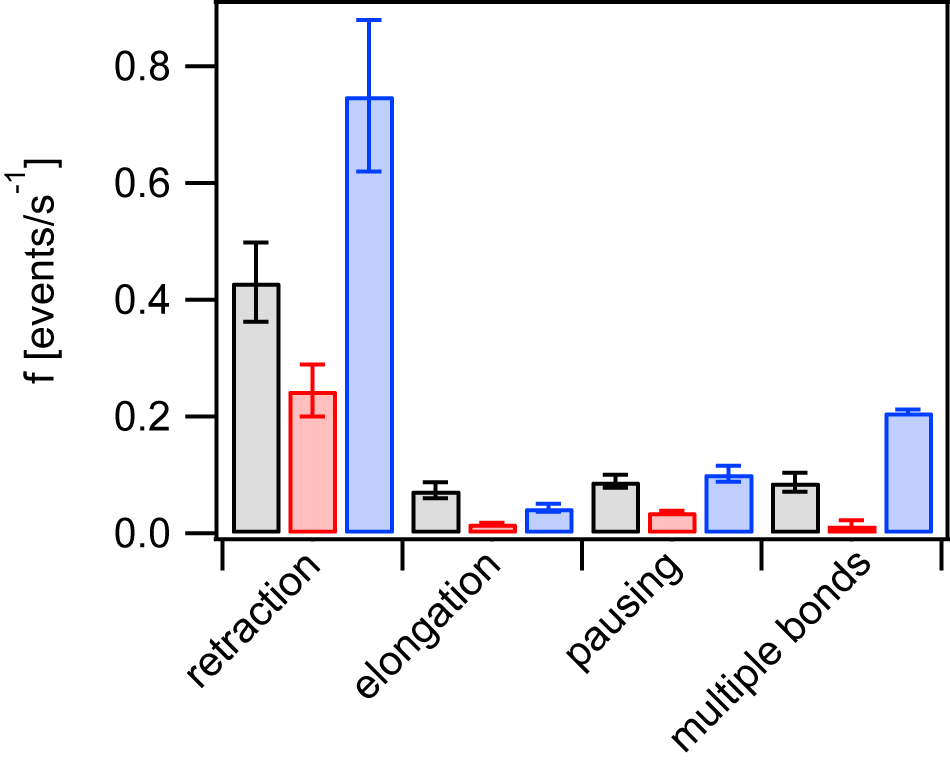

Supplement: S6 Fig — Grey: control, red: with 6.4 μg / ml azithromycin for (2–3) h, blue: with 0.4 μg / ml ceftriaxone for (2–3) h. Error bars: bootstrapping. (TIF) [file ppat.1009251.s006.tif]

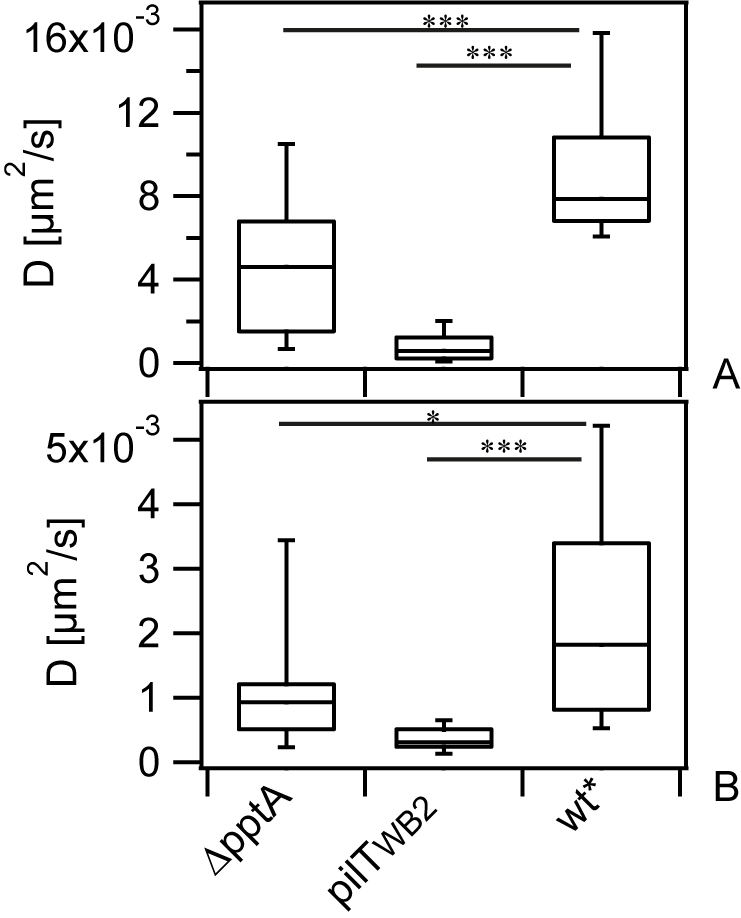

Supplement: S7 Fig — Mean effective diffusion constant D of untreated cells at A) the edge of the colony and B) within the colony at R = 5 μm after 6 h of growth for strains ΔpptA (Ng142), pilTWB2 (Ng176), and wt* (Ng150). Box: 25/75 percentile, whiskers: 10/90 percentile. N = 13–20. All statistical comparisons were made by two-sample KS-test: *P 0.05; ***P 0.001. (TIF) [file ppat.1009251.s007.tif]

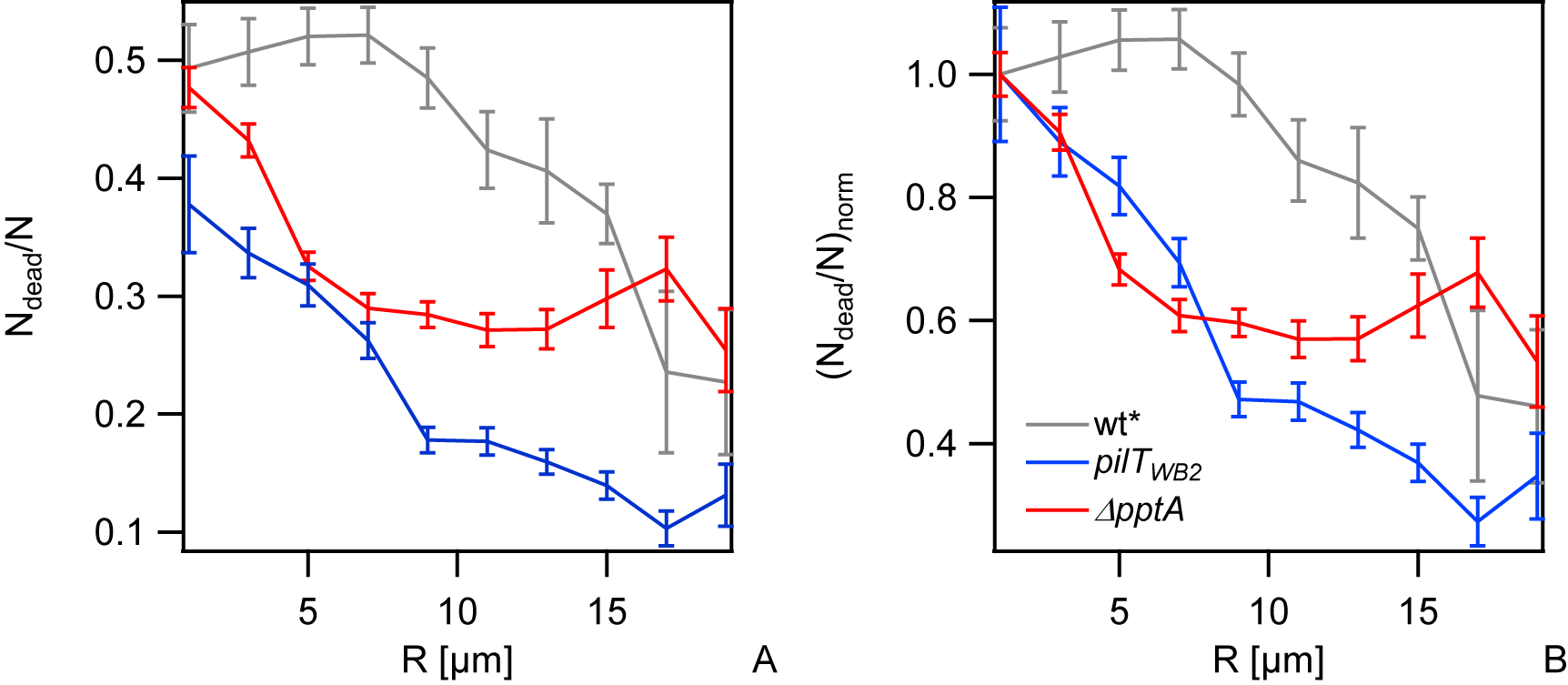

Supplement: S8 Fig — Bacteria were inoculated into flow chambers and colonies were allowed to assemble for 1 h. Subsequently, they were treated with with 0.4 μg / ml ceftriaxone for 5 h. A) Fractions of dead cells. B) Fractions of dead cells normalized to fraction at the edge. Grey: wt* (Ng150), blue: pilTWB2 (Ng176), red: ΔpptA (Ng142). Error bars: standard error of the mean. N = (9–46) colonies per data point. (TIF) [file ppat.1009251.s008.tif]

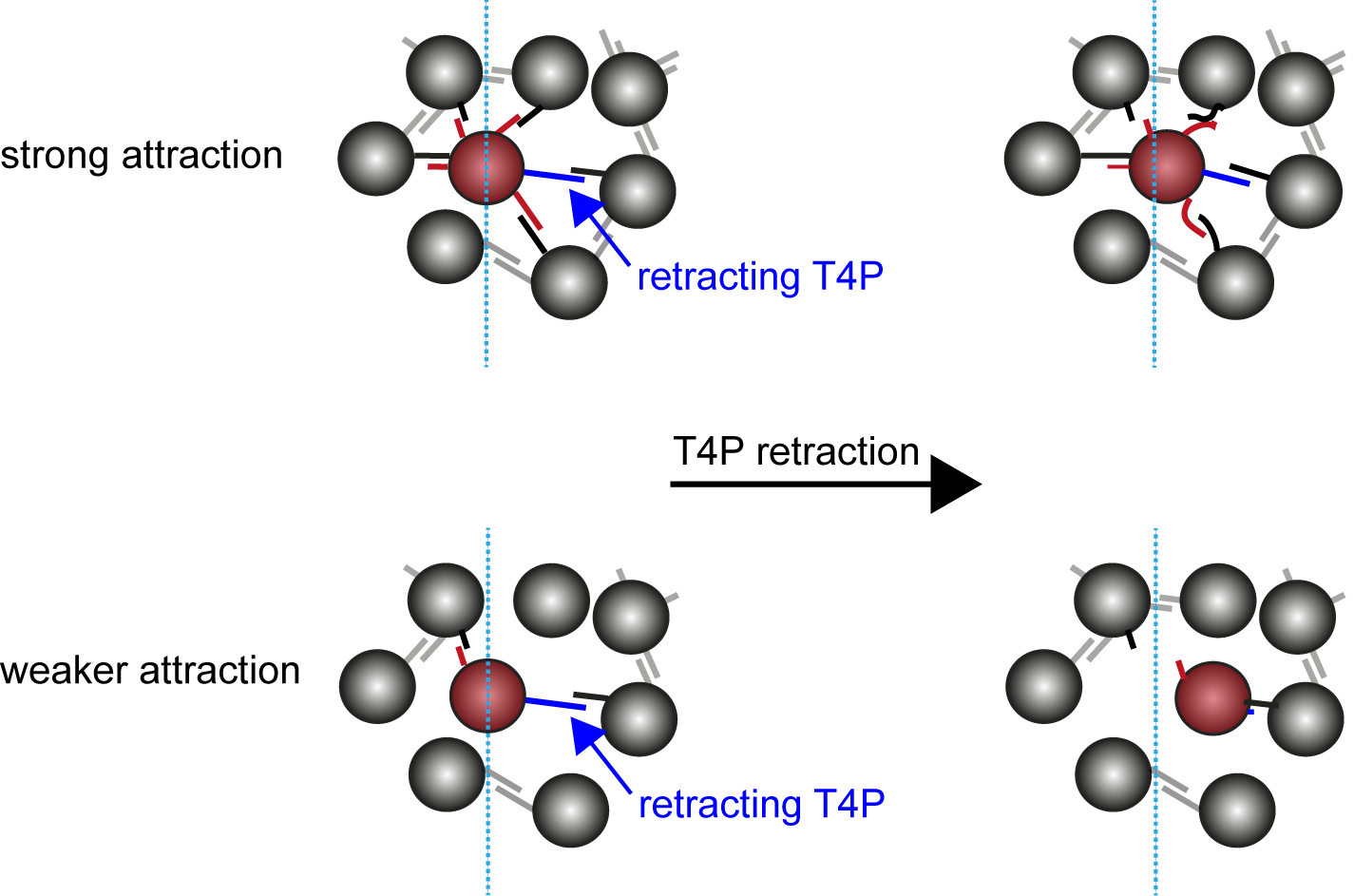

Supplement: S9 Fig — In the absence of azithromycin, a bacterium within the colony simultaneously forms multiple bonds with adjacent bacteria (top). When a T4P retracts, movement of the cell body is hindered by T4P-T4P bonds at the opposite side of the retracting pilus. In the presence of azithromycin, the probability that a T4P-T4P bond is formed is reduced (bottom). Thus, the probability that a retracting T4P has opposing T4P-T4P bonds is lower. As a consequence, the bacterium is more motile. (TIF) [file ppat.1009251.s009.tif]
