# Supplementary material for: Antibiotics modulate attractive interactions in bacterial colonies affecting survivability under combined treatment
Source: PLoS Pathog. 2021 Feb 1;17(2):e1009251. doi: 10.1371/journal.ppat.1009251 (PMC7877761; doi:10.1371/journal.ppat.1009251)
Supplement: S1 Table — (DOCX) [file ppat.1009251.s010.docx]

| **Strain** | **Relevant genotype** | **Source/Reference** |
| --- | --- | --- |
| *wt** (Ng150) | *G4::aac* | [17] |
| *pilT_WB2_* (Ng176) | *iga::P_pilE_ pilTWB ermC*  *G4::aac* | [16] |
| *ΔpptA* (Ng142) | *pptA::kan*  *G4::aac* | [17] |
| *ΔpilE* (Ng196) | *pilE::cat*  *G4::aac* | This study, [16] |
