# Supplementary material for: Antibiotics modulate attractive interactions in bacterial colonies affecting survivability under combined treatment
Source: PLoS Pathog. 2021 Feb 1;17(2):e1009251. doi: 10.1371/journal.ppat.1009251 (PMC7877761; doi:10.1371/journal.ppat.1009251)
Supplement: S2 Table — MICs were determined from bacteria that cannot form colonies (ΔpilE, Ng196) and from colony-forming wt* (Ng150) by testing for the ability to grow overnight. (DOCX) [file ppat.1009251.s011.docx]

| **antibiotic** | **MIC [µg / ml]**  *ΔpilE* | **MIC [µg / ml]**  wt* |
| --- | --- | --- |
| **azithromycin** | 0.064 | 0.128 |
| **ceftriaxone** | 0.004 | 0.008 |
| **ciprofloxacin** | 0.48 |  |
| **kanamycin** | 32 |  |
| **nitrofurantoin** | 0.002 |  |
| **trimethoprim** | 20 |  |
